# Prenatal and Postnatal Tobacco Exposure and Behavioral Problems in 10-Year-Old Children: Results from the GINI-plus Prospective Birth Cohort Study

**DOI:** 10.1289/ehp.0901209

**Published:** 2009-12-01

**Authors:** Simon Rückinger, Peter Rzehak, Chih-Mei Chen, Stefanie Sausenthaler, Sibylle Koletzko, Carl-Peter Bauer, Ute Hoffmann, Ursula Kramer, Dietrich Berdel, Andrea von Berg, Otmar Bayer, H.-Erich Wichmann, Rüdiger von Kries, Joachim Heinrich

**Affiliations:** 1 Ludwig-Maximilians University Munich, Institute of Social Pediatrics and Adolescent Medicine, Division of Epidemiology, Munich, Germany;; 2 Helmholtz Zentrum München – German Research Center for Environmental Health, Institute of Epidemiology, Neuherberg, Germany;; 3 Ludwig-Maximilians-University of Munich, Dr. von Hauner Children’s Hospital, Munich, Germany;; 4 Technical University of Munich, Department of Pediatrics, Munich, Germany;; 5 Institut für Umweltmedizinische Forschung, IUF, University of Düsseldorf, Düsseldorf, Germany;; 6 Marien-Hospital Wesel, Department of Pediatrics, Wesel, Germany

**Keywords:** adolescent health, behavioral problems, cohort study, environmental tobacco smoke exposure, strengths and difficulties questionnaire

## Abstract

**Background:**

Prenatal and postnatal tobacco exposure have been reported to be associated with behavioral problems. However, the magnitude of the association with tobacco exposure at specific periods of exposure is unclear.

**Objective:**

We assessed the relative risk of behavioral problems in children who had been exposed to tobacco smoke *in utero* and postnatally.

**Methods:**

We analyzed data from a prospective birth cohort study in two cities in Germany: the German Infant Nutrition Intervention. Our sample included 5,991 children born between 1995 and 1998 as well as their parents. We measured behavioral problems using the Strength and Difficulties Questionnaire (SDQ) at follow-up 10 years after birth. According to prespecified SDQ cutoff values, children were classified as “normal,” “borderline,” or “abnormal” according to the subscales “emotional symptoms,” “conduct problems,” “hyperactivity/inattention,” “peer-relationship problems,” and a total difficulties score. Smoke exposure and further covariates were assessed using parent questionnaires.

**Results:**

Compared with children not exposed to tobacco smoke, children exposed both pre- and postnatally to tobacco smoke had twice the estimated risk [95% confidence interval (CI), 1.4–3.1] of being classified as abnormal according to the total difficulties score of the SDQ at 10 years of age. Children who were only prenatally exposed had a 90% higher relative risk (95% CI, 0.9–4.0), whereas children who were only postnatally exposed had a 30% higher relative risk (95% CI, 0.9–1.9). These results could not be explained by confounding by parental education, father’s employment, child’s time spent in front of computer or television screen, being a single father or mother, or mother’s age.

**Conclusions:**

Prenatal exposure to tobacco smoke is associated with behavioral problems in school-age children. Although our findings do not preclude the influence of postnatal exposure, prenatal exposure seems to be more important.

Exposure of children to tobacco smoke, whether postnatal or *in utero*, is a well-known risk factor for various adverse health outcomes (DiFranza et al. 2004). An increased risk for intrauterine growth retardation, sudden infant death syndrome, and asthma are well-known adverse effects of *in utero* tobacco exposure (Higgins 2002). In addition, effects of *in utero* tobacco exposure on behavioral problems have been reported in various experimental and epidemiologic studies (Ernst et al. 2001; Eskenazi and Castorina 1999; Wakschlag et al. 2002; Weitzman et al. 2002) including some longitudinal studies (Markussen Linnet et al. 2006; Wakschlag et al. 1997). Prospective studies that systematically assess a broad range of behavioral problems outcomes are sparse.

Many studies have found an association between smoking in pregnancy and behavioral problems among children (Batstra et al. 2003; Roza et al. 2009; Saxton 1978). An independent effect of postnatal tobacco exposure on behavioral development has also been suggested (Braun et al. 2006, 2008; Fergusson et al. 1993; Weitzman et al. 1992; Williams et al. 1998). However, specifically delineating the impact of prenatal versus postnatal tobacco exposure is a challenging task: Children whose mothers have smoked during pregnancy are likely to be exposed to tobacco smoke after birth. Furthermore, many studies use cross-sectional designs where recall bias may play an important role. The specific roles of pre- and postnatal exposure are not yet clarified.

In this study, we analyzed data from the German Infant Nutritional Intervention (GINI), a large prospective birth cohort that also contains comprehensive follow-up. GINI’s prospective design and the comprehensive questions on tobacco smoke exposure at various time points provide the opportunity to disentangle the impacts of prenatal and postnatal tobacco exposure on behavioral problems. In a subset of this study, Gehring et al. (2006) tested the validity of the questionnaire-derived data on environmental tobacco smoke by measuring air nicotine and urine cotinine; the misclassification rate was below 7%, which is in line with that of other similar studies. We measured behavioral problems using the Strength and Difficulties Questionnaire (SDQ) (Goodman 1997), which allows assessment of a broad range of behavioral problems.

## Materials and Methods

### Study design

The GINI-plus study is an ongoing birth cohort study, initiated to prospectively investigate the influence of nutrition intervention during infancy plus the association of air pollution and genetics with the development of allergies. Between September 1995 and July 1998, a total of 5,991 healthy full-term newborns were recruited in obstetric clinics in Munich and Wesel, Germany. The number of mothers initially contacted was approximately 10,700, so the participation rate was 56%. The exclusion criteria of the study were severe acquired or congenital diseases, pregnancy of < 37 gestational weeks, birth weight < 2,500 g, or parents unable to complete the questionnaire. Children were followed up at the ages of 1, 2, 3, 4, 6, and 10 years. The cohort of children is composed of an intervention (*n* = 2,252) and a nonintervention group (*n* = 3,739). Group assignment was based on family history of allergy and willingness to participate in a randomized clinical trial. The intervention group received nutritional advice promoting breast-feeding for at least 4 months and participated in a randomized trial on the effect of hydrolyzed formula versus conventional cow’s-milk formula in preventing allergies (in the control group). The study protocol was approved by the local ethics committees (Bavarian General Medical Council, Medical Council of North Rhine Westphalia), and written consent was obtained from all participating families. Details on the study design are described elsewhere (Filipiak et al. 2007; von Berg et al. 2007).

### Definition of tobacco smoke exposure

Parents were asked for details on use of tobacco (“Have you smoked during pregnancy?”) at birth and at follow-up examinations when the child was 1, 2, 3, 4, 6, and 10 years of age. At each visit parents were asked about tobacco use over the preceding year (“In the last 12 months have you smoked in your home?”). If they had smoked, they were asked for the average number of cigarettes per day (or week). At the 10-year follow-up, parents were interviewed regarding exposure to smoking during the 7th, 8th, 9th, and 10th year of life of the child. Children were included in the analyses if their parents had participated and answered questions on smoking for at least 5 of 9 possible years. Children were defined as “never” being exposed to tobacco when the mother reported not smoking during pregnancy, and no smoking in the home was reported at follow-up. Children were classified as being only postnatally exposed to tobacco smoke when the parents reported that they had smoked in the home in at least one follow-up but the mother had not smoked during pregnancy. Pre- and postnatal exposure was defined for children whose mothers had smoked during pregnancy and smoking in the home was reported in at least one follow-up.

### Outcome measure

At the 10-year follow-up examination, the SDQ was completed by the parents (Goodman 1997; Goodman et al. 1998). The SDQ is an internationally applied and validated screening questionnaire (SDQ 2001). It assesses mental and behavioral difficulties and strengths of 3- to 16-year-old children along the following dimensions: “emotional symptoms,” “conduct problems,” “hyperactivity/inattention,” “peer-relationship problems,” and a “prosocial scale.” Each scale consists of five items, each rated on a three-point scale (“not true,” “somewhat true,” “certainly true”). Higher scores indicate more problems and/or more serious problems. In the present study, we used the German parent-reported version of the SDQ with the German cutoff points (Woerner et al. 2004). According to these cutoff points, children are classified as ”normal,” “borderline,” or “abnormal” on each scale and on the total difficulties score. These cutoff points were established in a representative sample of German children in order to classify about 10% of children as borderline and 10% as abnormal (Woerner et al. 2004).

### Statistical analysis

We dichotomized the results for the SDQ subscales, comparing children with abnormal SDQ scores with borderline and normal children. Results from each subscale and the total difficulties score were analyzed separately. We calculated Poisson regression models to obtain relative risk (RR) estimates. In these models age of mother at birth (< 30 years vs. ≥ 30 years), father’s employment [unemployed, not working, low to average-level work (such as clerk, craftsman, or public servant in intermediate service), high-level work (such as public servant in higher service, officer, or chief executive officer of a company)], and parental education (at least one parent with > 10 years of school education vs. both parents with < 10 years of school education), child’s time spent in front of computer or television screen (≥ 1 hr/day vs. < 1 hr/day), and being single father/mother were included as potential confounders. Furthermore the Poisson models included sex, study center, and intervention or nonintervention group as covariates. To obtain confidence intervals (CIs) for the RR estimates, we calculated robust error variance estimates as proposed by Zou (2004) using the GENMOD procedure with REPEATED statement in SAS (version 9.1; SAS Institute Inc., Cary, NC, USA).

### Potential dose effect

We assessed intensity of tobacco smoke exposure for the prenatal exposure in order to evaluate potential dose effects based on number of cigarettes smoked per day (“How many cigarettes were smoked in your home per day on average?”). Mothers were categorized as heavy smokers if they smoked > 5 cigarettes/day (median, 5 cigarettes/day) and as light smokers if they smoked < 5 cigarettes/day. In separate multivariable Poisson regression models, we calculated relative risks for being classified as abnormal in order to compare children of mothers who were heavy smokers with children of mothers who were light smokers and children who were never exposed to tobacco smoke (reference category).

## Results

A total of 2,862 children had complete information on smoking exposure at least at five follow-up time points plus SDQ assessment at 10 years of age. The dropout rate was considerably higher for children of young mothers and for children of parents with low level of education (Table 1). More than 90% of parents were classified as being highly educated, and > 50% of fathers worked in highly qualified jobs (Table 1). Because only very few fathers were unemployed or not working (e.g., still attending school), we excluded this group from the multivariable analyses and defined low- to average-level work as the reference category. The analyzed study population showed a higher proportion of subjects never exposed to tobacco smoke than subjects excluded from the study (Table 1).

At the 10-year follow-up, 6.3% (*n* = 181) of children were classified as having abnormal behavior and 5.8% (*n* = 167) were classified as borderline regarding the SDQ total difficulties score. The highest prevalence of abnormal and borderline behavior was observed for the subscale “conduct problems” (e.g., “Often lies or cheats” or “Steals from home, school or elsewhere”) where every fourth child (25.5%, *n* = 730) was outside the range of normal behavior. The lowest prevalence of abnormal or borderline behavior was observed for the SDQ subscale “hyperactivity/inattention” (e.g., “Easily distracted, concentration wanders” or “Restless, overactive, cannot stay still for long”); 5% (*n* = 143) were classified as borderline and 7.9% (*n* = 227) were classified as abnormal.

In multivariable Poisson regression models adjusting for potential confounders, exposure to tobacco smoke (both pre- and postnatal) was associated with behavioral problems measured by the SDQ (Table 2). The strength of the effect was dependent on the timing of tobacco smoke exposure. Children who were prenatally exposed to tobacco smoke had considerably higher risk of behavioral problems at 10 years of age, whereas postnatal exposure seemed to have only slight additional potential to cause problems. Postnatal exposure alone was also associated with abnormal behavior, but this association was weaker for most subscales of the SDQ (Table 2). The relative risk estimates for the impact of tobacco smoke exposure were comparable in the crude and adjusted analyses. For example, the relative risk for only prenatal tobacco exposure on total difficulties was 2.2 (95% CI, 1.1–4.6) in the crude analysis and 1.9 (95% CI, 0.9–4.0) in the adjusted analysis. These differences between the crude and the adjusted analyses were similarly small for other SDQ subscales (Table 2).

To assess a potential dose effect, we compared children of mothers who smoked heavily (> 5 cigarettes/day) during pregnancy with children of mothers who smoked lightly (< 5 cigarettes/day) during pregnancy and children never being exposed to tobacco smoke (reference category). The median number of cigarettes per day among smokers was 5. Unadjusted and adjusted relative risks are shown in Table 3. Children with heavy prenatal tobacco smoke exposure had consistently higher risks of abnormal behavior than did children with light exposure and children with no exposure.

As a sensitivity analysis, we also confined the multivariable analysis to children of parents with at least 10 years of education. In this analysis, the relative risks were comparable to the analysis on the total sample (data not shown).

## Discussion

In this prospective birth cohort with follow-up to 10 years of age, children who were exposed to tobacco smoke were found to be at increased risk for being classified as abnormal according to the SDQ relative to children with no tobacco exposure. The association seemed to be stronger for exposure during pregnancy. The association could not be explained by confounders such as parental education, father’s employment, child’s time spent in front of computer or television screen, being single father/mother, or age of the mother.

### Interpretation of the results

Prenatal exposure to tobacco smoke seemed to be the most influential exposure whether combined with postnatal exposure or not. Postnatal exposure alone seemed to have a lower impact on the risk for later abnormal behavior according to the SDQ, although fetal/placental/maternal metabolism may play a role in the differences between pre- and postnatal effect. Although prenatal exposure involves smoking by the mother only, postnatal exposure also includes smoking of the father in the household, which may dilute the effect of postnatal compared to prenatal exposure. Maternal smoking is more likely associated with intensive exposure to tobacco smoke than paternal smoking (Jaakkola and Jaakkola 2002). A diluted effect of postnatal exposure may partially explain the higher impact of prenatal exposure.

Among the SDQ subscales, one of the strongest associations of both prenatal and postnatal tobacco exposure was found for hyperactivity/inattention. This is in accordance with previous research from a cross sectional survey that used attention deficit hyperactivity disorder (ADHD) diagnosis as outcome measure (Braun et al. 2006). In that study, prenatal exposure to tobacco smoke was associated with later ADHD diagnosis, whereas postnatal exposure at home was not (Braun et al. 2006). Furthermore, studies on the effects of *in utero* tobacco exposure have shown more pronounced effects on externalizing problems than on internalizing problems (Batstra et al. 2003; Gatzke-Kopp and Beauchaine 2007; Yolton et al. 2009).

### Possible biological mechanisms

Tobacco smoke contains various toxic substances, and there are several possible links to fetal neurotoxicity. Neurotoxicity is hypothesized to occur via *a*) hypoxic effects on the fetal–placental unit (reduction of fetal blood flow, toxic increase in carbon monoxide) and *b*) teratologic effects on the developing nervous system (Wakschlag et al. 2002). The fetal brain is protected against many neurotoxicants—but not against nicotine, which crosses the placental barrier and acts as a neurotransmitter on nicotinic receptors (Koren 1995). Although specificity is problematic, given the complexity of the central nervous system, the pattern of SDQ changes—namely, the higher hyperactivity/inattention and conduct problems scores—is suggestive: Continuous patterns of higher nicotine levels can alter noradrenalin and dopamine response (Sharma and Brody 2009); the current physiological hypothesis on ADHD is a relative shortage of noradrenalin and dopamine (Brennan and Arnsten 2008). Animals exposed to prenatal nicotine and carbon monoxide showed hyperactivity and deficits in arousal modulation (Ajarem and Ahmad 1998; Thomas et al. 2000; Tizabi et al. 1997). Another possible explanation is the potential indirect effects of tobacco exposure. Smoking is associated with lower birth weight and perinatal problems, all of which increase the risk for later antisocial behavior. Given the sample of participants analyzed, healthy full-term births, these factors should not play an important role in the investigated sample.

### Strengths and limitations

The SDQ is not a diagnostic instrument but only a screening test. However, we feel that this is a strength of our study. Children of parents with low socioeconomic status are more likely to have ADHD but are less likely to be diagnosed and treated as having ADHD (Froehlich et al. 2007). The SDQ relies only on perceptions of the parents and not on potential differential access to health care.

We have combined postnatal exposure into one single variable (postnatal tobacco exposure, yes/no). However, there may be more and less relevant postnatal time periods for the impact of tobacco smoke. Unfortunately the numbers in our analyses were not large enough to accurately compare, for example, children with only early postnatal exposure or only late postnatal exposure. However, we feel that the approach of disentangling the prenatal from the postnatal tobacco exposure should not be affected by this limitation.

We used self-reports of smoking which might underestimate the true exposure to tobacco smoke. Furthermore, prenatal exposure relates only to active smoking of the mother. However, these limitations should not significantly affect the interpretations of our results. For example, it may be plausible that women who have children with behavioral problems tend to conceal smoking. This could lead to an underestimation of the association between smoke exposure and behavioral problems. If the mother’s passive exposure to environmental tobacco smoke in pregnancy is associated with behavioral problems, this would also lead to an underestimation of the effects. Therefore, regarding potential exposure misclassification, we believe that underestimation of the effects is possible.

Because of incomplete information or loss to follow-up, a high number of cases had to be excluded from the analyses, [*n* = 3,129 (52.2%)] which may be associated with potential bias. Children of young mothers and less-educated parents and children exposed to tobacco smoke were more likely to be excluded. Although children of mothers who smoked were more likely to be lost to follow-up, we do not feel that this influenced the association between smoke exposure and behavioral problems. If “difficult” children of mothers who smoked had been less likely to be followed up, this would account for an underestimation of the observed association. When we confined the analysis to children of parents with at least 10 years of education, the results were comparable to those in the total sample.

The association we found could not be explained by confounding by parental education, father’s employment, or age of mother. Adjusting for these factors should warrant a relatively reliable consideration of socioeconomic status of the parents. Because socioeconomic status is both likely associated with smoking and with behavioral problems, this is crucial. Nevertheless, residual confounding cannot be excluded. However, the study group was relatively homogeneous in socioeconomic status because of the sample design, which included only healthy newborns of German nationality. The majority of parents were relatively highly educated, and more than half of fathers worked in highly qualified jobs. Therefore we feel that confounding by socioeconomic status is unlikely to explain our results. However, potential confounding by parental psychological problems could not be addressed and cannot be excluded.

## Conclusions

In a prospective cohort study of considerable size we were able to demonstrate a strong association of *in utero* exposure to smoking with several dimensions of abnormal behavioral development as assessed by the SDQ, an established and standardized test. Our data gave no indication for similarly strong associations with only postnatal exposure to tobacco smoke.

## Figures and Tables

**Table 1:** Characteristics of the GINI study population including 5,991 children born 1995–1998 in Wesel (North Rhine–Westfalia, Germany) and Munich (Bavaria, Germany) [no. (%)].

Characteristic	Study population analyzed (*n* = 2,862)	No complete follow-up (*n* = 3,129)*a*
Sex		
Male	1,456 (50.9)	1,158 (52.5)
Female	1,406 (49.1)	1,046 (47.5)
Center		
Wesel	1,372 (47.9)	1,670 (53.4)
Munich I	727 (25.4)	679 (21.7)
Munich II	763 (26.7)	780 (24.9)
Tobacco smoke exposure		
Never	1,569 (54.8)	435 (47.1)
Only postnatal	900 (31.5)	329 (35.6)
Only prenatal	69 (2.3)	26 (2.8)
Pre- and postnatal	324 (11.3)	134 (14.5)
Father’s employment		
Unemployed or not working	94 (3.3)	68 (4.1)
Low to average level	1,263 (44.1)	788 (47.7)
High level	1,505 (52.6)	795 (48.2)
Mother’s age at birth (years)		
≥ 30	1,955 (68.3)	1,747 (56.1)
< 30	907 (31.7)	1,367 (43.9)
Parental education (years)		
At least one with > 10	2,630 (91.9)	2,485 (82.8)
No parent with > 10	232 (8.1)	515 (17.2)
Single mother/father		
Yes	293 (10.2)	73 (18.8)
No	2,569 (89.8)	316 (81.2)
Time in front of screen		
< 1 hr/day	1,895 (66.2)	275 (65.8)
≥ 1 hr/day	967 (33.8)	143 (34.2)

a These numbers may not sum to 3,129 because of missing data.

**Table 2:** Unadjusted and adjusted RRs for the association between pre- and postnatal tobacco smoke exposure and abnormal SDQ values at 10 years of age.

Behavioral problem	Crude RR (95%CI)	Adjusted RR*a* (95%CI)
Total difficulties score		
Tobacco smoke exposure		
Never (72)	1	1
Only prenatal (7)	2.2 (1.1–4.6)	1.9 (0.9–4.0)
Only postnatal (59)	1.4 (1.0–2.0)	1.3 (0.9–1.9)
Pre- and postnatal (36)	2.5 (1.7–3.6)	2.0 (1.4–3.1)

Hyperactivity		
Tobacco smoke exposure		
Never (97)	1	1
Only prenatal (5)	1.2 (0.5–2.8)	1.1 (0.5–2.7)
Only postnatal (74)	1.3 (1.0–1.8)	1.2 (0.9–1.6)
Pre- and postnatal (42)	2.1 (1.5–3.0)	1.8 (1.3–2.6)

Emotional symptoms		
Tobacco smoke exposure		
Never (140)	1	1
Only prenatal (11)	1.8 (1.0–3.1)	1.5 (0.9–2.8)
Only postnatal (83)	1.0 (0.8–1.4)	1.0 (0.7–1.3)
Pre- and postnatal (44)	1.5 (1.1–2.1)	1.4 (1.0–1.9)

Peer problems		
Tobacco smoke exposure		
Never (119)	1	1
Only prenatal (6)	1.1 (0.5–2.5)	1.1 (0.5–2.4)
Only postnatal (84)	1.2 (1.0–1.6)	1.2 (0.9–1.5)
Pre- and postnatal (38)	1.6 (1.1–2.2)	1.4 (1.0–2.0)

Conduct problems		
Tobacco smoke exposure		
Never (158)	1	1
Only prenatal (12)	1.7 (1.0–2.9)	1.6 (0.9–2.8)
Only postnatal (104)	1.2 (0.9–1.5)	1.1 (0.9–1.5)
Pre- and postnatal (61)	1.9 (1.4–2.5)	1.8 (1.3–2.3)

Numbers in parentheses after the smoke exposure categories indicate the number of children in each category who had abnormal SDQ values.

a Adjusted for for sex, study center, intervention group, parental education, father’s employment, age of mother at birth, child’s time in front of screen, and being single father/mother.

**Table 3:** Unadjusted and adjusted RRs for the association between intensity of prenatal tobacco smoke exposure and abnormal SDQ values at 10 years of age.

Behavioral problem	Crude RR (95%CI)	Adjusted RR*a* (95%CI)
Total difficulties score		
Tobacco smoke exposure		
Never (72)	1	1
Low prenatal (3)	2.0 (0.7–6.0)	1.4 (0.4–4.8)
High prenatal (3)	3.0 (1.0–8.8)	2.6 (1.0–7.1)

Hyperactivity		
Tobacco smoke exposure		
Never (97)	1	1
Low prenatal (1)	0.5 (0.1–3.4)	0.4 (0.1–3.0)
High prenatal (3)	2.2 (0.8–6.5)	1.9 (0.6–6.0)

Emotional symptoms		
Tobacco smoke exposure		
Never (140)	1	1
Low prenatal (3)	1.0 (0.3–3.0)	0.8 (0.3–2.7)
High prenatal (6)	3.1 (1.6–6.2)	2.9 (1.4–5.9)

Peer problems		
Tobacco smoke exposure		
Never (119)	1	1
Low prenatal (3)	1.2 (0.4–3.6)	0.9 (0.3–3.1)
High prenatal (2)	1.2 (0.3–4.6)	1.1 (0.3–3.7)

Conduct problems		
Tobacco smoke exposure		
Never (158)	1	1
Low prenatal (5)	1.5 (0.7–3.4)	1.3 (0.5–3.0)
High prenatal (4)	1.8 (0.8–4.5)	1.6 (0.7–3.8)

Numbers in parentheses after the smoke exposure categories indicate the number of children in each category who had abnormal SDQ values. Children never exposed serve as a reference category. The numbers of children with prenatal exposure only and abnormal SDQ values may not sum to the respective number in Table 2 because of missing information on numbers of cigarettes smoked. Low exposure, < 5 cigarettes/day; high exposure, > 5 cigarettes/day.

a Adjusted for for sex, study center, intervention group, parental education, father’s employment, age of mother at birth, child’s time in front of screen, and being single father/mother.
